# Extracellular Vesicles and Intercellular Communication: Challenges for In Vivo Molecular Imaging and Tracking

**DOI:** 10.3390/pharmaceutics15061639

**Published:** 2023-06-01

**Authors:** Debora Petroni, Costanza Fabbri, Serena Babboni, Luca Menichetti, Giuseppina Basta, Serena Del Turco

**Affiliations:** 1 Institute of Clinical Physiology, CNR San Cataldo Research Area, Via Moruzzi 1, 56124 Pisa, Italy; 2 Institute of Life Sciences, Scuola Superiore Sant’Anna, 56127 Pisa, Italy

**Keywords:** cell–cell communication, extracellular vesicles, endothelial dysfunction, cardiovascular disease, biodistribution, molecular imaging

## Abstract

Extracellular vesicles (EVs) are a heterogeneous class of cell-derived membrane vesicles released by various cell types that serve as mediators of intercellular signaling. When released into circulation, EVs may convey their cargo and serve as intermediaries for intracellular communication, reaching nearby cells and possibly also distant organs. In cardiovascular biology, EVs released by activated or apoptotic endothelial cells (EC-EVs) disseminate biological information at short and long distances, contributing to the development and progression of cardiovascular disease and related disorders. The significance of EC-EVs as mediators of cell–cell communication has advanced, but a thorough knowledge of the role that intercommunication plays in healthy and vascular disease is still lacking. Most data on EVs derive from in vitro studies, but there are still little reliable data available on biodistribution and specific homing EVs in vivo tissues. Molecular imaging techniques for EVs are crucial to monitoring in vivo biodistribution and the homing of EVs and their communication networks both in basal and pathological circumstances. This narrative review provides an overview of EC–EVs, trying to highlight their role as messengers of cell–cell interaction in vascular homeostasis and disease, and describes emerging applications of various imaging modalities for EVs visualization in vivo.

## 1. Introduction

The inter-cellular and inter-organ communication systems are crucial both in the maintenance of tissue homeostasis and in disease development [1]. Cell-to-cell communication involves soluble factors such as metabolites, lipids, proteins, and extracellular vesicles (EVs) that can be transferred from the parent cell to other cells at short or long distances [2].

EVs are a heterogeneous class of cell-derived membrane vesicles released by various cell types and can be classified according to their size and their biogenesis into three main classes: exosomes, microvesicles, and apoptotic bodies. Widely distributed in diverse tissues and body fluids, EVs emerge as an additional mechanism for delivering biomolecules and signals to neighboring or distant target cells influencing recipient cells’ biology [3,4]. Increasing evidence suggests that EVs’ effects on target cells mainly depend on cargo content transferred by EVs, which often differs from the pathophysiological conditions of the host cells. In fact, depending on the condition of the source cells, EVs shuttle a wide range of lipids, proteins, metabolites, messenger RNA (mRNA), and microRNA that trigger a cascade of signaling pathways in recipient cells, preserving tissue homeostasis or inducing pathological changes [4,5].

The endothelium is a metabolically active organ of biological importance for maintaining homeostatic function. Continuous damage of the vascular endothelium leads to endothelial activation and apoptosis, the development of endothelial dysfunction, and subsequent atherosclerotic lesion formation, all processes involved in the pathogenesis of cardiovascular diseases (CVDs) [6,7]. In these conditions, EVs released by endothelial cells (EC–EVs) constitute a large subclass of all circulating EVs in peripheral blood that contribute to the pathogenesis of atherosclerosis, myocardial infarction, and other vascular disorders [8].

Thus, the study of functional characterization and distribution of EC–EVs elicits considerable interest not only as biological markers to monitor and treat CVDs but also as therapeutic tools for the targeted delivery of therapeutics.

Despite the significant progress regarding the role of EC–EVs as mediators of cell–cell and inter-organ crosstalk, a complete understanding of how local and distal intercommunication plays in physiological and pathological conditions is lacking. Most of the studies performed on EVs derive from isolated cell cultures, co-cultures, or a mixture of EVs isolated from human body fluids, but there are still little reliable data available on biodistribution and specific homing of EVs in vivo tissues. Once released in circulation, EVs may reach neighboring cells and, potentially, distant organs, transfer their cargo, and act as mediators of intercellular information. Molecular imaging techniques for in vivo tracking of EVs are crucial to understand the mechanism of uptake and to monitor the in vivo biodistribution and homing of EVs and their communication networks in basal and pathological conditions. These tools will also allow the development and optimization of EV-based diagnosis and treatment.

In this review, we provide an overview of EVs of endothelial origin as messengers of intercellular communication in vascular homeostasis and disease. Moreover, we present and discuss current and emerging applications of various imaging modalities for EV visualization that will be able to facilitate a better understanding of their biodistribution, biological functions, and their potential as therapeutics and drug delivery vehicles.

## 2. EVs Biogenesis and Classifications

**Figure 1:**
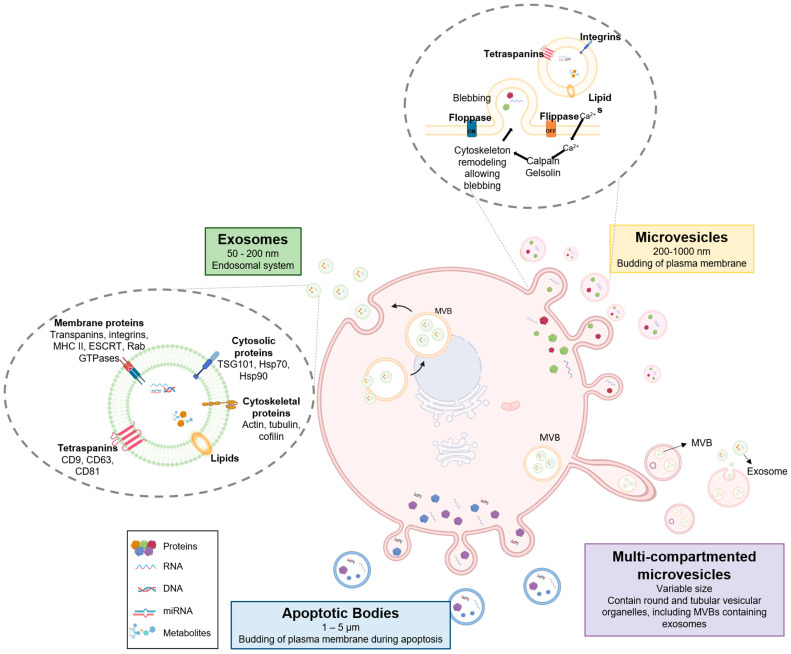
Sketch of biogenesis and characteristics of EVs. The EVs differ in size and biogenesis. Exosomes or small EVs (50–200 nm) derive from the inward budding endosomal multivesicular bodies. Microvesicles or medium EVs (200–1000 nm) are formed and secreted directly from the plasma membrane’s outward budding. A new mechanism has been now identified in endothelial cells that leads to the release of multi-compartmented microvesicles from the protrusion of cell membrane. Apoptotic bodies are an additional type of microvesicle formed when a cell undergoes programmed cell death. ESCRT: endosomal sorting complex required for tran; GTPase: guanine nucleotide-binding proteins (G-proteins); Hsp: heat shock proteins; MCH: major histocompatibility complex; MVBs: endosomal multivesicular bodies; TSG101: tumor susceptibility gene 101.

EVs are ubiquitously released by almost every cell type, and consequently, they have been identified in body fluids, including blood, urine, saliva, breast milk, cerebrospinal fluid, bronchoalveolar lavage fluid, synovial fluid, semen, and amniotic fluid [9]. EVs are released under physiological conditions but also upon cellular activation, senescence, and apoptosis. During EV biogenesis, they are loaded with various cellular components, including DNA, coding and noncoding RNAs (mRNA, miRNA, circRNA, tRNA), mostly miRNAs, lipids and proteins, and surface receptors associated with the EV membrane. Their contents depend on the parent cell, the microenvironment, and the triggers preceding their release [4]. After fusion with the cell membrane, EVs are released into the extracellular space and play the role of intercellular mediators by transferring their cargo between cells in living organisms [10,11]. EVs are classified based on their biological properties: biogenesis pathways, size, and biomarkers (Figure 1). EVs can be mainly classified based on their size in small (<100 nm or <200 nm) and medium/large EVs (>200 nm) [12].

Exosomes or small EVs (50–200 nm) derive from the inward budding of the internal multivesicular compartments, named endosomal multivesicular bodies (MVBs) [13,14]. Exosomes are highly enriched in tetraspanin protein able to organize membrane nanodomains related to cell adhesion and migration [15]. Their cone-shaped tertiary structure, together with their ability to associate with transmembrane receptors and to bind to cytoskeletal and signaling scaffolds, allows tetraspanins to regulate endosomal network dynamics and exosome biogenesis and cargo selection [15]. CD9, CD63, and CD81 are some tetraspanin proteins found enriched in exosomes, often used as EV surface markers [15,16]. Membrane proteins in exosomes, besides transpanins, include MHC class II complexes; an endosomal sorting complex required for transport (ESCRT); integrins [17]; membrane-associated molecules such as Rab GTPases; cytoskeletal proteins including actin, tubulin, and cofilin; and cytosolic proteins including ALG-2-interacting protein X (Alix), tumor susceptibility gene 101 (TSG101), and heat shock proteins (e.g., Hsp70 and Hsp90) [18,19]. Furthermore, exosome cargo consists of lipid, protein, DNA, and coding and noncoding RNAs (mRNA, miRNA, circRNA, tRNA), but mostly miRNAs.

Microvesicles or medium EVs (>200 nm) are formed and secreted directly from the plasma membrane’s outward protrusion or budding [20,21]. Microvesicles are released, especially during cell growth or following cell activation, by proinflammatory stimuli, hypoxia, oxidative stress, etc. [21]. The formation and release of MVs are initiated by an increase in cytosolic calcium, which activates two proteases: the calpain, which removes membrane proteins from the intracellular cytoskeleton [22], and the gelsolin bound to actin filaments [23]. The cleavage of the actin protein network leads to cytoskeleton remodeling, allowing blebbing.

In the resting cell, phosphatidylserine is located in the inner leaflet of the lipid bilayer of the plasma membrane. When the cell is activated, the increase in cytosolic calcium also activates the enzyme floppase, which allows the movement of plasma membrane lipids to the outer membrane. In this way, phosphatidylserine is exposed on the outer leaflet of the phospholipid bilayer and can be easily detected as it binds to annexin V [24]. In a recent study, Peterson et al. described microvesicles containing multiple membrane compartments (referred to as multi-compartment microvesicles or MCMVs) that originate from MVB-containing cell protrusions at specialized sites on the surface of endothelial cells. MCMVs contain MVBs that can release exosomes after transiting away from the parent cell. The packaging in multiple layers of membranes is thought to protect the cargo from being degraded in the extracellular space and being able to travel farther before being released by MCMV or taken up into recipient cells. Multiple membrane layers could also help cargoes avoid cytoplasmic lysosomal degradation in the recipient cell and reach the nucleus [25].

The largest EVs (1–5 μm) are apoptotic bodies that are produced from apoptotic cells when they undergo programmed cell death [26].

Each subtype of EVs should be expected to have distinct molecular characteristics and functions. However, the lack of standard protocols for isolating EVs based on their size and density does not allow for the production of homogeneous populations of each subtype. The recognition of these limitations in EV isolation has led the International Society for EVs (ISEV) to recommend the use of “EV” as a broad classification term for these vesicle types [12,27].

This review focuses mainly on exosomes and microvesicles. Since certain studies have not specifically analyzed the subtype of the vesicle, and many of these terms are protocol-dependent and relative, we refer to the general term EVs in place of the names used in the original literature to avoid terminology ambiguity.

## 3. Endothelial EVs: An Example of Intercellular Communication in CVD

The endothelium, situated at the interface of the blood vessel lumen and wall, integrates physical and humoral signals derived from blood and surrounding tissue to regulate the vascular tone and maintain an anti-inflammatory and antithrombotic surface [6,28]. EVs released by endothelial cells participate in local and systemic communication and are implicated both in the maintenance of vascular homeostasis and in various diseases involving endothelial injury or dysfunction [29,30]. EC–EVs carry a rich cargo, whose composition reflects the parent cell’s characteristics, capable of mediating phenotypical changes to neighboring cells, contributing to the circulating secretome, and reaching distant sites where exacerbate systemic endothelial injury [31,32]. In physiological conditions, the fraction of EVs secreted by both ECs and endothelial progenitor cells is relatively low and mainly promotes vascular homeostasis in a paracrine way. EC–EVs contain anti-inflammatory miRNAs that modulate vascular inflammation [33], mRNAs that promote angiogenic program [34], or antioxidant enzymes that contribute to antioxidant defense in oxidative stress conditions such as aging [35]. Conversely, low shear stress and inflammatory and apoptotic stimuli have been also shown to elicit a great number of EVs with proinflammatory and prothrombotic characteristics [36,37,38] that can act as a causal and contributing factor in altering vascular cell phenotype. High levels of EC–EVs have been found in patients with coronary artery disease (CAD) [39], acute coronary syndrome [40], and myocardial infarction [41], representing a mechanism responsible for the dissemination of the procoagulant and proinflammatory potentials to sites remote from the microenvironment of their formation.

The dysfunctional vascular endothelium is a driver of atherogenesis [42], and some studies demonstrated the role of EC–EVs in atherosclerosis progression and related disorders [43,44], while others suggested atheroprotective properties [33,45].

A recent study identified EC–EVs containing miRNA as markers of cell–cell communication in atherosclerotic vasculature. Atherogenic ECs can transfer miR-92a to macrophages via EVs inducing macrophage inflammation, migration, and lipoprotein uptake in a Krüppel-like factor 4 (KLF)4-dependent manner and contributing to the atheroprone macrophage phenotype and subsequent lesion progression [46]. The miR-92a has also been found in the endothelial EVs isolated from the plasma of CAD patients, and in vitro experiments have also demonstrated that EC–EVs containing miR-92a-3p promote angiogenesis, likely acting as a regenerative signal to improve myocardial function by increasing blood flow to ischemic areas of the heart [45]. Both in vitro and in vivo models have reported that EC–EVs stimulate angiogenesis through the delivery of miRNA-214 to neighboring ECs [47].

The phenotype switching of vascular smooth muscle cells (VSMC) plays a critical role in the pathogenesis of several cardiovascular diseases, including hypertension and atherosclerosis. EC–EVs enriched in miR-143/145 can act as mediators of intercellular communication between ECs and VSMCs exerting atheroprotective effects. The endothelial overexpression of Krüppel-like factor 2, an important mediator of the anti-inflammatory and anti-thrombotic properties, induces the release of EVs containing miR-143/145 that prevent VSMC proliferation and migration, key events of atherosclerotic plaque development [48]. Additionally, the intravenous administration of KLF2-expressing endothelial EVs reduced atherosclerosis in an in vivo model [48].

Endothelial dysfunction and VSMC plasticity are critically involved in the pathogenesis of hypertension and arterial stiffness, a consequence of atherosclerosis. EC dysfunction induced by Angiotensin II results in the release of EVs containing miR-92a, whose serum levels are high in hypertensive patients, to the neighboring VSMCs modulating the contractile-to-proliferative phenotypic change of VSMCs, which leads to arterial stiffness [49]. 

A recent study has demonstrated that VSMC-derived EVs isolated from diabetic patients promote EC and macrophage activation in a miR-221/222 dependent manner. Furthermore, the administration of these EVs promotes increased atherosclerotic plaque formation in the ApoE^−/−^ mouse model of atherosclerosis, representing a novel paracrine signaling pathway in the cardiovascular complications of diabetes [50].

An example of EV-mediated organ communications is described between EVs derived by circulating blood cells and endothelial cells. Platelet- and monocyte-derived EVs represent the main regulators of the endothelium, affecting vasodilation, leukocyte recruitment, apoptosis, and thrombosis. Platelet-derived EVs are the most abundant type of vesicle found in circulation under physiological conditions and play a role in maintaining endothelial function [51]. Furthermore, it has been demonstrated that platelet-derived EVs can induce endothelial growth factor angiogenesis and stimulate post-ischemic revascularization following chronic ischemia [52]. Platelet-derived EVs affect EC activation inducing the expression of adhesion molecules, allowing the adhesiveness of circulating immune cells to the vessels [53]. After platelet activation in vitro, EVs containing miR-223 impair the endothelial barrier function restraining contractility [54]. Moreover, EV platelet-derived miR-142-3p delivered into ECs induces EC apoptosis in an in vivo hypertensive rat model [55].

The communication between EC–EVs and monocyte-derived EVs plays an active role in vascular inflammation that, in turn, can lead to CVDs [33]. Circulating monocytes are components of innate immunity, and inflammatory conditions facilitate their adhesion and migration to the vascular endothelial wall, promoting atherosclerotic lesion formation. EVs released by activated monocytes can promote EC thrombogenicity, activation and interaction with leukocytes [56], and apoptosis [57], acting as active mediators of endothelial dysfunction and inflammation state [58].

EV-mediated crosstalk among ECs and cardiomyocytes (CMs) is of the utmost importance in maintaining normal heart function, promoting cardiac repair, and maintaining heart metabolic requirements under stress conditions. It has been demonstrated that EVs produced from human CMs are preferentially taken up by ECs, rather than fibroblasts or cardiomyocytes [59]. Intramyocardial injection of EVs secreted by ischemic cardiomyocytes confers protection against oxidative stress-induced lesions and induces neovascularization, likely contributing to sustaining cardiac repair following injury [60]. In conditions of glucose deprivation, CM–EVs increase the proliferation and tube formation capacity of ECs and stimulate increased glucose uptake, glycolytic activity, and pyruvate production in cardiac microvascular ECs in vitro [61]. Moreover, CM–EVs isolated from a rat model of type 2 diabetes inhibit the angiogenic properties of cardiac ECs through the exosomal transfer of miR-320 [62].

EC–EVs isolated from obese/hypertensive adults induced a significantly higher expression of hypertrophic and fibrotic proteins in CMs, suggesting their role as a potential mediating factor in the increased risk of cardiomyopathy and heart failure with obesity/hypertension [63].

Additionally, ECs can transmit protective signals to cardiomyocytes by EVs. In fact, EVs released by normoxic ECs are able to protect cardiomyocytes from hypoxia/reoxygenation injury through the activation of the ERK1/2 pathway [64].

These studies underlie that EC–EVs, as mirrors of endothelial dysfunction, can play a role as messengers in CVD and in related disorders. Since data are obtained from co-culture systems of EV-donor and EV-recipient cells, it is also important to prove biodistribution and cell uptake of EC–EVs but, in general, of all EVs, in in vivo models.

## 4. Molecular Imaging: A Tool to Study EV Crosstalk

Molecular processes concerning the composition and behavior of EVs are commonly studied using in vitro approaches. However, for a better understanding of the cell–cell communication networks mediated by EVs and their dynamics [65], it is essential to refer to a pertinent context that considers the effects of the physiological microenvironment [66].

Recent advances in technologies and EV-labeling strategies allowed the investigation of the behavior of EVs directly in living organisms, in a non-invasive way [66], thus overcoming all limitations associated with in vitro studies that provide “static” results obtained in isolated settings [67].

In order to understand the involvement of EV crosstalk in physiological processes, the more suitable strategy is the targeting of endogenous EVs in vivo, which allows investigation of the dynamics of EVs from production to uptake in recipient cells without any external interference [65]. Verweij et al. adopted this approach to track exosomes in vivo in zebrafish embryos by expression of the CD63-pHluorin reporter, a pH-sensitive fluorophore that quenches in acidic cell organelles, to assess the secretion, journey, and fate of endogenous EVs in real-time, demonstrating the functional inter-organ communication [68].

The study of endogenous EVs is tricky [69] and only partially translatable to mammalian models [70,71]; therefore, the most common approach for studying the biodistribution of EVs in vivo is injecting exogenous vesicles, isolated from conditioned cell culture media or biological fluids (plasma, urine, spinal fluid, saliva, etc.) [69].

Tracking EVs in living organisms is a challenge due to their natural origin, small size, and short half-life [72]. The possibility of monitoring in vivo the trafficking of exogenously introduced EVs permits collecting a series of information (tissue half-life, excretion pathways, etc.) needed to deepen the still incomplete knowledge about their dynamics [67] and for their potential application in the therapeutical/drug delivery field.

Molecular imaging (MI) represents one of the more suitable approaches to investigate the fate of labeled exogenous EVs and to live track inter-organ communication. This is a powerful tool able to visualize in real-time biochemical events at the cellular and molecular level within living cells, tissues, and/or intact subjects in a non-invasive way [73]. Preclinical MI includes different techniques: optical imaging (fluorescence and bioluminescence), nuclear imaging (positron emission tomography and single photon emission computed tomography), magnetic resonance, computed tomography, and photoacoustic [73]. The characteristics of the different imaging modalities, summarized in Table 1, are fundamental in choosing the most suitable MI technique for in vivo EV tracking.

In order to perform clinical or, in large animals, preclinical studies, techniques with an adequate depth of penetration, such as SPECT, PET, CT, or MRI, will be suitable. For the detection of very low concentrations, a remarkable sensitivity is necessary; hence, the choice may fall on optical or nuclear imaging. When a high spatial resolution is requested, FLI and BLI are not among the most suitable techniques.

**Figure 2:**
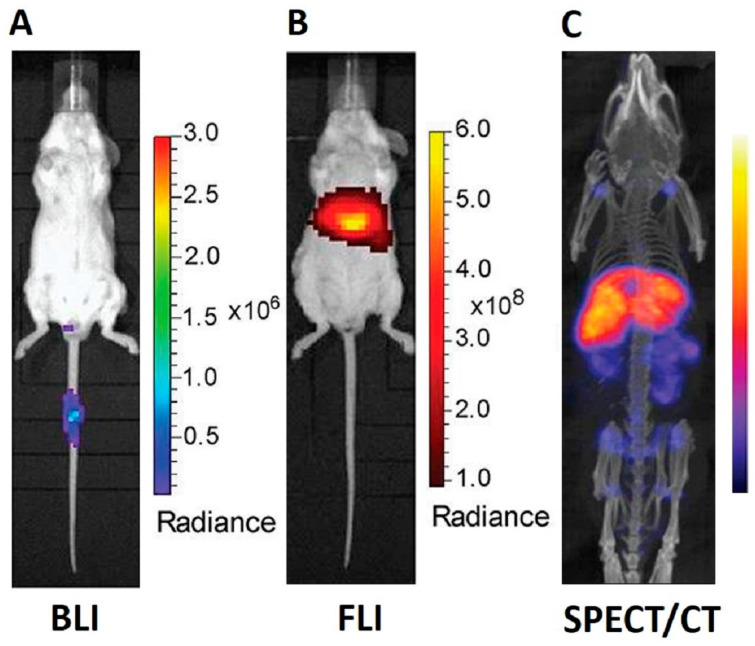
Examples of visualization of EVs in vivo using BLI, FLI, and SPECT/CT imaging. Representative images of subcutaneous CT26 tumor-bearing BALB/c mice at 24 h post-intravenous injection with the same dose of EVs, obtained from human embryonic kidney cells (Expi293F), engineered with CD63-NanoLuc Luciferase (Nluc) for bioluminescence imaging (**A**), labeled with DiR for fluorescence imaging (**B**), and radiolabeled with ^111^In-DTPA for SPECT/CT imaging (**C**). All panels are adapted and taken from [74].

To highlight the different types of visualization resulting from each modality, Figure 2 shows images obtained by BLI, FLI, and SPECT/CT imaging of labeled EVs produced from the same cell source and injected to BALB/c tumor-bearing mice systemically with the same dose and administration route [74]. From the comparison of the images, it is evident how SPECT/CT imaging (panel C) is a technique able to provide more accurate and easily readable pictures.

The choice of the most appropriate technique should therefore be considered carefully, weighing up pros and cons according to the nature of the process to investigate.

**Figure 3:**
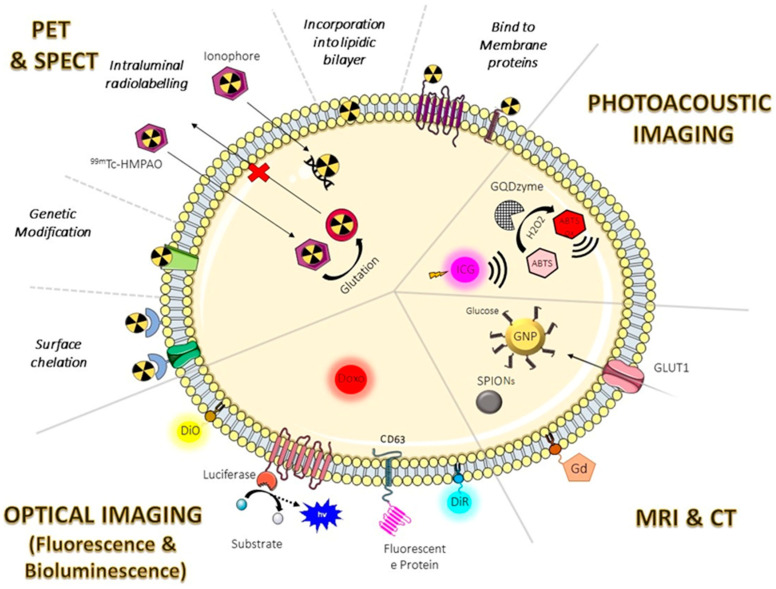
Schematic representation of labeling methods of EVs. CT: Computed tomography; GQDzyme: Graphene quantum dot nanozyme; GNP: Gold nanoparticles; Glut1: Glucose transporter 1; Gd: Gadolinium; GBCA: Gadolinium (Gd)-based paramagnetic contrast agents; HMPO: Hexamethylpropyleneamine oxime; MRI: Magnetic resonance imaging; PET: positron emission tomography; SPECT: single photon emission computed tomography; SPIONs: Superparamagnetic iron oxide nanoparticles.

For each imaging modality, targeted probes have been developed using different strategies of EV labeling, as outlined in Figure 3, where a schematic picture of the different methods is outlined [75,76,77].

### 4.1. Fluorescence Imaging (FLI)

Fluorescence imaging is based on the detection of the light emitted from some molecules (fluorophores) following absorbing electromagnetic radiation. It is a safe imaging method (radiation-free) and it is reasonably simple to use, both in terms of time and money. The key benefits of this technique are high labeling efficiency and good sensitivity (10^−9^–10^−12^ M) and signal-to-noise ratio. Despite this, fluorescence imaging has disadvantages, including a low depth of penetration (<10 mm) caused by light attenuation by tissues and low resolution (2–3 mm). The resolution of optical imaging is optimal only for ex vivo analyses of tissues or for studies in vivo in small transparent animals such as zebrafish embryos [67].

Fluorescence is among the most widely used techniques for tracking EVs. This method is based on the use of lipophilic dyes, fluorescent protein reporters, and encapsulation or binding of fluorescent compounds, as shown in Figure 3 [77].

Several lipophilic dyes, able to provide consistent fluorescence signals for both in vitro and in vivo observation, are commercially available. The membrane of EVs is easy to stain directly with these dyes since its unilamellar lipid bilayer is similar to that of cells. The most common, carbocyanine dyes (DiO, DiL, DiR, DiD, etc.), frequently used to label lipids on the plasma membrane or in liposomes, were also used for labeling EVs [77]. The problems correlating with the use of lipophilic dyes are the formation of aggregates and micelles and the presence of unbound dyes, non-labeled EVs, and the long half-life [78], which may generate artifacts [79]. 

Fluorescent protein reporters are usually introduced into mother cells and combined with EV membrane proteins. The sequence of the gene encoding the EV marker protein (e.g., CD9, CD63, and CD81) is fused with the fluorescent protein gene for this purpose. To produce fluorescent EVs, this gene is inserted into EV-producing cells using viral or plasmid vectors [80]. However, these techniques may alter the parent cells’ properties, and these modifications may also affect the EVs [81].

Another method of labeling consists of incorporating fluorescent molecules into the intraluminal space of EVs. For example, Ingato et al. loaded Doxorubicin inside nanovesicles and exploited its red fluorescence emission properties to study the in vivo biodistribution of vesicles loaded with the chemotherapeutic [82].

An alternative to tracking EVs with a constant fluorescent signal is to use sensitive probes with a variable fluorescent signal. These probes are able to produce a variable signal as a function of changes in the surrounding environment (e.g., pH changes [83] or following gene editing tools [70,84]). This method allows the monitoring of the secretion, cellular uptake, and fate of endogenous EVs, but it is not suitable for exogenous vesicles [85].

### 4.2. Bioluminescence Imaging (BLI)

Bioluminescence consists of the emission of photons generated by natural processes; more specifically, by the chemical reaction between the luciferase enzyme and a substrate. This imaging technique has the benefit of having a high sensitivity (10^−15^–10^−17^ M), extremely low background emission, and not requiring an external source of stimulation to produce light. In addition, since the auto-luminescence in mammalian tissue is very low, it guarantees a far higher signal-to-noise ratio than fluorescence [81].

Despite that, the use of bioluminescent technology has several significant drawbacks. It is not able to provide anatomical context; therefore, it should be combined with MRI or CT. In addition, the quick signal fading, the low penetration capacity (10–20 mm), and poor spatial resolution (3–5 mm) further restrict the traceability of bioluminescence [67]. The optical signals must be generated through substrate injection, and this represents another drawback. These substrates might be harmful to animals, and repeated injection might cause problems for long-term sequential imaging [86].

For bioluminescence imaging, bioluminescent reporter protein genes must be transduced into the mother cells. Thus, EVs produced by these cells will express the protein within the vesicle or on the membrane (indirect labeling). However, this strategy, in addition to being time-consuming, can alter the normal activity of transduced cells [87]. Using bioluminescence imaging, EVs may be seen in a highly accurate and dependable manner, and due to the modest size of the label, incorporation into EVs is less likely to have a substantial impact [67]. However, there are difficulties associated with the genetic labeling of transmembrane proteins; in fact, it can interfere with ligand–receptor interaction or cause steric hindrance and organotropism [88,89,90].

Among the luciferases most commonly used for labeling EVs, there is Renilla luciferase (RLuc) [91] gLuc-LA, produced by the fusion of Lactadherin (a tropic protein of exosomes) with the reporter Gaussia luciferase (gLuc) [92], and ThermoLuc, identified as the best for in vivo studies [93].

### 4.3. Nuclear Imaging (PET, SPECT)

Nuclear imaging techniques include positron emission tomography (PET) and single photon emission computed tomography (SPECT), which are based on the use of probes containing gamma or positron-emitting radionuclides. Both PET and SPECT are frequently utilized for molecular imaging due to their many benefits, such as their high labeling effectiveness, large field of view, superior sensitivity (10^−10^–10^−11^ M), contrast agent specificity, outstanding temporal resolution (seconds to minutes), and high deep-tissue penetration (allowing whole-body scanning). Despite its many advantages, the high cost and the need for suitable facilities limit the use of nuclear imaging [72]. Nuclear imaging can offer data for a full knowledge of the in vivo behavior of EVs and can be used in conjunction with other technologies, such as MRI and CT, to better localize EVs (as shown in Figure 2C) [77]. Radiolabeling does not change the characteristics of EVs significantly; regardless of the radionuclides used, no appreciable changes were observed in the size, morphology, zeta potential, or markers of EVs after labeling [94,95,96,97].

There are two primary methods for radiolabeling EVs: surface and intraluminal, as shown in Figure 3. Surface or membrane radiolabeling is the most common method of radiolabeling EVs, which can involve direct labeling, the incorporation of the radionuclide by genetic modification, the incorporation into the membrane, and the labeling by a chelator (with a metal-binding moiety for sequestering radiometals) [97]. The most often used radionuclide for surface tagging are isotopes of iodine (^123^I, ^124^I, ^125^I, and ^131^I) and Tecnetium-99m. ^131^I-labeled EVs from endothelial progenitor cells were used to assess the differential in vivo distribution in metastatic breast cancer animal models by SPECT [98].

Technetium-99m was also used as a complex in the form of both [^99m^Tc(CO)_3_(H_2_O)_3_]^+^, able to bind several amino acids of the surface proteins [76], and [^99m^Tc]Tc-Annexin-V-128, able to bind the phosphatidylserine molecules present in the membrane of EVs. The latter was used for the study of the in vivo biodistribution of EC–EVs after systemic injection in a preclinical model of hind limb ischemia, demonstrating an early and specific homing of radiolabeled EC–EVs to the ischemic hind limb with therapeutic angiogenic effects [99].

The radionuclides Copper-64, Gallium-68, Zirconium-89, and Indium-111 require the presence of a bifunctional chelator for binding to the surface of EVs [97]. A different strategy for radiolabeling EVs is the intra-vesicular space capturing of the radiotracer (intraluminal). Methods used to enable radionuclide internalization in EVs include remote loading and ionophore-chelating binding. The first uses the ability of intravesicular endogenous glutathione to convert [^99m^Tc]Tc-hexamethylpropyleneamine oxime ([^99m^Tc]Tc-HMPAO) from the lipophilic to hydrophilic form [100] and trap it in the aqueous core of the vesicles. The second method exploits the formation of lipophilic complexes between ionophore ligands (such as tropolone and 8-hydroxyquinoline) and radionuclides, allowing transport across the phospholipid membrane, and, once inside, the radionuclide is released and binds the intraluminal proteins and nucleic acids [101].

### 4.4. Magnetic Resonance Imaging (MRI)

MRI is a widespread imaging technique both in biomedical research and the clinical field [67] whose advantages include the high spatial resolution and depth of penetration (Table 1), the long half-life of the contrast agents, and the ability to provide detailed anatomical images due to high soft-tissue contrast. The main drawback of MRI is a low sensitivity (10^−3^–10^−5^ M) that requires high concentrations of contrast agents to have a sufficient signal.

In order to be located by MRI, EVs must be carrying a magnetic contrast agent; Gadolinium (Gd)-based paramagnetic contrast agents (GBCA) are the most widely used MRI-positive contrast agent and have also been used to label EVs. The incorporation of Gd into EVs is performed by the conjugation of phospholipids. The surface charge, size distribution, and/or shape of the EVs were slightly altered after labeling, although the protein markers and size of the EVs remained unchanged. This alteration could be due to the extrusion process used to insert the Gd-based agent into the membranes of the EVs [102,103,104].

Superparamagnetic iron oxide nanoparticles (SPIONs) are also used to label EVs (Figure 3). Hu et al. encapsulated the SPIONs into melanoma cell-derived exosomes by electroporation and demonstrated, for the first time, how EVs loaded with this cargo are suitable to be tracked by MRI [105].

### 4.5. Computed Tomography Imaging (CT)

CT imaging is based on the detection of X-ray beams and their reprocessing to obtain a three-dimensional picture of analyzed tissue. It enables ultrafast, real-time scanning with excellent penetration power and high spatial resolution (50–200 µm). The main disadvantages of CT imaging are exposure to ionizing radiation and poor resolution of soft-tissue anatomical images [67].

Gold nanoparticles (GNPs) are the most frequently used probes in CT imaging. Due to their superior X-ray absorption and bio-inertness, they are also widely used for imaging EVs.

Betzer et al. [106] used glucose-coated GNPs to track EVs in the brain. The glucose coating allowed them to exploit a GLUT-1-mediated mechanism, allowing gold nanoparticles to be inserted inside EVs simply by incubation (Figure 3). The characterization of these nanoparticles showed that gold labeling had no discernible impact on EV size or size distribution; however, more research is required to determine the impact of gold nanoparticles.

Glucose-modified gold nanoparticles were also used to label EVs derived by mesenchymal cells. EVs were visualized by in vivo CT imaging to determine the biodistribution after injection into a myocardial infarction mouse model, revealing their retention in the myocardial infarction area and their usefulness for improving heart function [107].

### 4.6. Photoacoustic Imaging (PAI)

The imaging technique known as photoacoustic tomography combines ultrasonic imaging and photoacoustic effect. Because PAI enables non-invasive, real-time imaging and has the benefit of high spatial resolution (100–500 µm), high contrast, and good depth of penetration (<50 mm), it is a particularly alluring imaging modality [108]. Additionally, there is no radiation exposure danger, and it is less expensive than CT and MRI.

Few studies have been conducted on the tracking of EVs using PAI. Ding et al. developed an exosome-like nanozyme vesicle for the H_2_O_2_-responsive PAI of nasopharyngeal carcinoma. In the presence of H_2_O_2_, the graphene quantum dot nanozyme (GQDzyme), which has intrinsic peroxidase activity, successfully transforms 2,2′-azino-bis(3-ethylbenzothiazoline-6-sulfonic acid) (ABTS) into its oxidized form [109]. The oxidized ABTS is a suitable contrast agent for PAI since it has a strong near-infrared (NIR) absorbance. Nasopharyngeal cancer cells, which emit H_2_O_2_ in response to laser radiation, could be tracked by the nanoparticles produced [109]. In another recent study, indocyanine green (ICG) was used as a contrast agent. The ICG was loaded inside the EVs and incubated with paclitaxel and sodium bicarbonate for therapeutic chemo-sonodynamic combination purposes with pH-responsive PA imaging guidance [110] (Figure 3).

### 4.7. Multimodal Imaging

Multimodal imaging consists of the intriguing combination of two or more imaging techniques, allowing the production of multiple signals simultaneously and, thus, multiple information for the same sample, particularly when the modalities involved have complementary advantages. For multimodal imaging, the EVs have to be labeled with two or more probes suitable for the different imaging modalities involved.

Jung et al., developed a new multimodal method to monitor the biodistribution of EVs in vivo. EVs extracted from tumor cells were successfully labeled with SCN-NOTA-(^64^Cu or ^68^Ga) and the Cy7 fluorescence dye and were visualized by PET and optical imaging [111]. Santos-Coquillat et al. designed a dual-sEV probe based on SPECT and fluorescent imaging to track goat milk EVs using ^99m^Tc(IV) and sulfo-cyanine5 NHS ester (SCy5) [112].

Lv et al. [113] used the dual-modality PAI/MRI to investigate the behavior of tumor-derived cellular microvesicles labeled with Gd-based melanin nanoparticles, while Lai et al., used FLI/BLI imaging to assess the biodistribution of EVs realized by combining Gaussia luciferase and metabolic biotinylation [114].

## 5. Tracking EVs In Vivo: Issues and Challenge

Although imaging represents an extremely useful tool for the in vivo investigation of EVs pharmacokinetics [66], only a few publications regarding these approaches are reported in the literature.

A systematic review of the literature of Kang et al. about studies of the biodistribution of EVs in living organisms following administration has highlighted only 38 publications matching the search criteria, and among these, only six regarding the biodistribution of EVs by in situ imaging [97]. In the following years, several works collecting the application of imaging for the study of EVs were published [77,115,116,117]; what emerges is an extremely heterogeneous set of information involving varying imaging methodologies, goals to achieve, types of used EVs, etc. [118]. Some studies aimed to track the uptake of EVs in a specific organ, others targeting tumors. The only element in common is the animal model. Most experiments were performed on mice and indicated the liver, spleen, and sometimes lungs and kidneys as the organs with the highest accumulation of EVs [118]. Recently, other animal models, i.e., rats [119], non-human primates (*Macaca nemestrina)* [120], and zebrafish, were used to study the pharmacokinetics and biodistribution of injected EVs [121].

Results obtained from experiments performed on zebrafish make it a very promising model to track the behavior of endogenous or exogenous EVs in vivo and understand their role in inter-organ communication [65,83,121,122]. In addition, zebrafish represent an ideal model to investigate endogenous EV functions in multiple different cardiovascular functions and pathologies [123]. Indeed, using a transgenic membrane-tethered fluorophore reporter system, Scott A. et al. demonstrated that EVs can be released by multiple cell types, including ECs and CMs, in larval and adult zebrafish in vivo and that EVs are transferred between different cell types in the adult zebrafish heart. EC–EVs were observed within intravascular macrophages, potentially suggesting targeted crosstalk between ECs and cardiac macrophages [124].

Zebrafish have emerged as a powerful model organism in research because they present many advantages, including high fecundity, rapid external development, and unrivaled cellular level in vivo imaging, and stable transgenic zebrafish lines expressing cell membrane-tethered fluorophores would also label EVs produced by those cells. The high percentage (more than 70%) of orthologous genes in common with Homo sapiens, the easy management, and the low cost with respect to rodents [125] represent additional advantages associated with the use of this specimen to be considered a versatile pre-mouse model [69]. Despite that, the use of this animal model involves some difficulties [69], especially when the use of the adult model and more sophisticated imaging techniques are requested. The limiting factor is the technological equipment [125] since tomographic scanners are not suitable for the imaging of fish, although numerous studies are currently underway to overcome this obstacle [126,127].

Among the MI modality previously described, fluorescence is currently the most used technique to study in vivo EV biodistribution [128], with the advantages and drawbacks previously discussed. However, in order to translate the preclinical approach to a clinical environment, the use of other MI techniques with higher sensitivity and depth of penetration is requested. Nuclear imaging techniques, combined with CT to obtain anatomical information about EV localization [72], are an excellent tool to visualize and understand inter-organ communications. Although PET and SPECT imaging are very expensive and require suitable facilities, they allow for obtaining very precise information about the journey of injected EVs and quantifying their concentration in the organ/tissue under investigation.

The lack of standardization in the research and the difficulties associated with the use of vesicles greatly complicate this type of study [65]. The variability observed in published works, combined with a disagreement on nomenclature and sometimes an insufficient description of the experimental details that hindered the reproducibility of the study [65], makes it difficult to find a common thread.

Many aspects and critical issues have to be taken into account before approaching an in vivo study, starting from preliminary isolation steps (e.g., ultracentrifugation), which can lead to heterogeneity in the subpopulation [77,129], but also to various modifications of EVs, such as loss of functional integrity, aggregation, and deformation [130]. In fact, one critical issue that needs to be understood is the effect of membrane engineering of both EV membranes and cargo on the biophysical, functional, and pharmacological properties of EVs. In particular, it remains to be determined how altering the membrane composition affects the lipid composition of the EVs and whether it affects EV function or absorption. Several key aspects of engineered EVs, such as stability, tropism, and release kinetics, might also require further investigation.

In addition, it is known that the biodistribution of EVs is affected by dosage, route of administration, and cellular origin [131], as well as by the labeling process, which can modify their characteristics [118]. Lazaro-Ibanez et al., comparing different technologies and, therefore, the tag of the relative probe to use, indeed demonstrated how the labeling strategy affects the sensitivity of the detection and tracking of EVs [74].

All these aspects have to be thorough and need to be improved in order to better understand the EV-mediated inter-organ cross-talk in vivo [69].

## 6. Conclusions

In the regulation of cardiovascular biology, EC–EVs mediate vascular signaling by transferring their cargo into neighboring and, possibly, distant recipients, resulting in detrimental and favorable effects depending on the status of both the cellular origin and the recipient cell. Studies show that EC–EVs can contribute both to atherosclerosis development and progression and vascular homeostasis, suggesting the role of EC–EVs in the diagnostic and therapeutic approach to various diseases.

The involvement of EVs in the intercellular signaling network is now a fact. What requires more knowledge are the mechanisms of cell–cell communication; data provided by EV biodistribution studies are a pivotal connecting link in the investigation of vesicle behavior as they represent the bridge between in vitro results and physiological dynamics. Molecular imaging represents a useful approach to evaluate the fate of EVs in vivo since it allows monitoring in real-time the trafficking of labeled EVs. Nevertheless, in order to fully exploit the potential of this tool, all problems that still make the investigation of EVs trafficking in vivo difficult must be solved. The intrinsic properties of EVs (size, half-life, etc.), the difficulties and technical issues for their isolation (separation methods, subpopulation heterogeneity, etc.), the lack of standardized protocols for their characterization, and the poor reproducibility of the experimental procedures reported in the literature are aspects that must necessarily be improved and consolidated. Further critical issues emerge from the labeling of EVs for their application as probes for molecular imaging. The labeling methods must be developed so as not to significantly change the properties of the EVs (sizes, activity, etc.), have to be assessed with proper quality controls, and the probes obtained have to be stable and with a half-life compatible with the time required to complete the study under investigation.

Future activities must therefore be focus on the set-up of standardized protocols, validated methods and controls, in order to overcome current limitations and deeply understand EVs dynamics in living organisms.

## Figures and Tables

**Table 1:** Properties of molecular imaging modalities.

Modality	Temporal Resolution	Spatial Resolution in Clinical	Spatial Resolution in Pre-Clinical	Sensitivity	Depth of Penetration
FLI	Seconds–minutes	-	2–3 mm	10^−9^–10^−12^ M	<10 mm
BLI	Seconds–minutes	-	3–5 mm	10^−15^–10^−17^ M	10–20 mm
SPECT	Minutes	8–10 mm	1–2 mm	10^−10^–10^−11^ M	Unlimited
PET	Seconds–minutes	5–10 mm	1–2 mm	10^−10^–10^−12^ M	Unlimited
MRI	Minutes	0.25–1 mm	25–100 µm	10^−3^–10^−5^ M	Limited by magnet bore
CT	Minutes	0.5–1 mm	50–200 µm	ND	Unlimited
PAI	Seconds–minutes	10 µm–1 mm	100–500 µm	ND	<50 mm

FLI: Fluorescence imaging; BLI: Bioluminescence imaging; SPECT: Single photon emission computed tomography; PET: Positron emission tomography: MRI: Magnetic resonance imaging; CT: Computed Tomography imaging; PAI: Photoacoustic imaging; ND: Non-detectable.

## Data Availability

Not applicable.
